# Enamel Integrity and Residual Composite Following Clear Aligner Attachment Removal: A Systematic Review

**DOI:** 10.3390/clinpract16030051

**Published:** 2026-02-27

**Authors:** Nicolas Nassar, Karim Corbani, Rim Bourgi, Roland Kmeid, Carlos Enrique Cuevas-Suárez, Ahmed A. Holiel

**Affiliations:** 1 Department of Orthodontics, Faculty of Dental Medicine, Saint-Joseph University of Beirut, Beirut 1107 2180, Lebanon; nicolas.nassar.dds@hotmail.com (N.N.); rolandkmeid@gmail.com (R.K.); 2 Craniofacial Research Laboratory, Faculty of Dental Medicine, Saint-Joseph University of Beirut, Beirut 1107 2180, Lebanon; 3 Department of Digital Dentistry, AI and Evolving Technologies, Saint-Joseph University of Beirut, Beirut 1107 2180, Lebanon; 4 Department of Restorative and Esthetic Dentistry, Faculty of Dental Medicine, Saint-Joseph University of Beirut, Beirut 1107 2180, Lebanon; karim.corbani@usj.edu.lb; 5 Laser Unit, School of Dentistry, Saint-Joseph University of Beirut, Beirut 1107 2180, Lebanon; 6 Department of Biomaterials and Bioengineering, INSERM UMR_S 1121, University of Strasbourg, 67000 Strasbourg, France; 7 Dental Materials Laboratory, Academic Area of Dentistry, Autonomous University of Hidalgo State, San Agustín Tlaxiaca 42160, Mexico; 8 Department of Conservative Dentistry, Faculty of Dentistry, Alexandria University, Alexandria 5424041, Egypt; ahmed.holiel@alexu.edu.eg; 9 Department of Restorative Sciences, Faculty of Dentistry, Beirut Arab University, Beirut 115020, Lebanon

**Keywords:** clear aligner, debonding, enamel loss, orthodontic attachment, profilometry, residual composite, surface roughness

## Abstract

Objectives: This systematic review evaluated enamel surface alterations and residual composite following the removal of clear aligner attachments, with particular emphasis on the influence of removal techniques, instrument selection, operator experience, and the use of magnification on enamel preservation and cleaning efficiency. Methods: A comprehensive electronic search was performed in PubMed, Scopus, Embase, Web of Science, and Scielo up to October 2025. In vitro, ex vivo, and clinical studies assessing enamel loss, residual composite, surface roughness, or removal time after clear aligner attachment removal were included. Study selection, data extraction, and methodological assessment followed the PRISMA 2020 guidelines and Cochrane Handbook recommendations. Risk of bias was evaluated using a modified Joanna Briggs Institute checklist for laboratory-based studies. Due to substantial methodological heterogeneity, a narrative synthesis was conducted. Results: Of 656 identified records, three in vitro/ex vivo studies were assessed for eligibility. Reported enamel loss ranged from approximately 15 µm to more than 50 µm, depending on the removal protocol and visualization conditions. Residual composite covered approximately 20–40% of the treated enamel surface. Multi-step protocols combining tungsten carbide burs with silicone polishers under magnification demonstrated the most favorable balance between composite removal efficiency and enamel preservation. Fiberglass burs were associated with smoother enamel surfaces but increased enamel loss, whereas one-step polishing systems (OneGloss, Enhance, SM104) resulted in reduced surface roughness and shorter procedural time. The use of magnification loupes (≥2.5×) consistently improved removal precision and reduced residual composite. Meta-analysis was not feasible due to heterogeneity in outcome measures and testing methodologies. Overall risk of bias was deemed acceptable. Conclusions: Based on the limited number of available in vitro/ex vivo studies, removal of clear aligner attachments appears to be associated with measurable enamel loss and residual composite, largely influenced by the instruments and visualization aids used. Sequential carbide–silicone polishing protocols performed under magnification appear promising based on limited in vitro/ex vivo evidence, demonstrating a favorable balance between composite removal and enamel preservation under controlled laboratory conditions. However, given the scarcity of evidence and absence of clinical trials, these findings cannot be directly extrapolated to routine clinical practice. Further well-designed studies are required before definitive clinical recommendations can be established.

## 1. Introduction

Clear aligner therapy has emerged as a mainstream orthodontic treatment, offering enhanced aesthetics, patient comfort, and compliance compared with traditional fixed appliances [1,2,3]. The clinical efficacy of aligners depends not only on the design of the thermoplastic shells but also on auxiliary tools known as composite attachments, which are bonded to the teeth to control and optimize force application during tooth movement [2,4]. These attachments are fabricated using composite resins of varying viscosities, ranging from low-viscosity flowable nanocomposites for better adaptation to condensable composites that provide superior mechanical strength and resistance to functional stresses [4].

Upon completion of orthodontic treatment, the removal of composite attachments represents a critical clinical step. The primary objective is to restore the enamel surface as closely as possible to its pre-treatment condition, preserving long-term dental health, aesthetics, and resistance to demineralization [5]. However, this procedure presents inherent challenges. Improper removal can lead to iatrogenic enamel damage, including increased surface roughness, microcracks, grooves, or enamel loss, while residual composite may serve as a nidus for biofilm accumulation, staining, or secondary caries. The risk and severity of these outcomes are influenced by the type of composite used, the instrumentation, and the technique applied during attachment removal [6].

A wide array of methods has been proposed to remove residual composite after orthodontic procedures. Commonly employed tools include tungsten carbide burs, zirconia burs, fiber-reinforced composite burs, white stone burs, and aluminum oxide-based polishing systems [7,8,9,10]. While tungsten carbide burs are favored for their efficiency, studies have reported enamel damage even when polishing instruments are employed in conjunction [9,11]. Conversely, multi-step polishing systems and one-step aluminum oxide-based devices have demonstrated improved enamel preservation, though their performance varies depending on grit size, material composition, and operator technique [12,13]. Additionally, laser-assisted approaches have recently emerged, offering selective ablation of composite material while potentially minimizing enamel damage and enhancing procedural precision [6].

Assessment of enamel integrity after composite removal relies on both qualitative and quantitative methods. Scanning electron microscopy (SEM) allows high-resolution visualization of surface morphology [11,14,15,16], while profilometry [13,17,18,19], atomic force microscopy (AFM), and non-contact three-dimensional (3D) optical techniques [20,21] provide objective measures of surface roughness and topography. Recent advances, such as micro-computed tomography (micro-CT) and 3D scan superimposition [22], enable precise quantification of enamel loss and volumetric evaluation of residual composite, offering a comprehensive framework to evaluate procedural outcomes.

Despite extensive research on adhesive removal following bracket debonding, the unique clinical scenario of clear aligner attachments remains underexplored. Unlike bracket removal, which often applies mechanical force directly to the enamel, attachment removal involves thicker, flowable composites molded onto the tooth surface, generating subtle overflow that is challenging to visualize and remove. Consequently, standard protocols developed for bracket debonding may not adequately mitigate enamel damage or residual composite in the context of aligners. This gap in the literature underscores the need for a systematic, evidence-based appraisal of attachment removal methods specific to clear aligner therapy. However, the available evidence remains scarce and is currently limited to laboratory-based investigations, reflecting the emerging nature of this field. Therefore, this systematic review aims to critically evaluate the available evidence regarding enamel surface alterations and residual composite following clear aligner attachment removal, with particular emphasis on the influence of removal techniques, instrument selection, operator-related factors, and visualization aids. Given the limited and exclusively in vitro/ex vivo evidence base, the findings should be interpreted with caution. Ultimately, the goal is to provide guidance that balances operational efficiency with maximal preservation of enamel integrity, enhancing both the safety and effectiveness of aligner treatment outcomes.

## 2. Materials and Methods

### 2.1. Study Design

This systematic review was conducted in accordance with the Preferred Reporting Items for Systematic Reviews and Meta-Analyses PRISMA 2020 guidelines and the Cochrane Handbook for Systematic Reviews of Interventions (Table S1) [23]. The review protocol was prospectively registered on the Open Science Framework (Registration: https://osf.io/a42wm, accessed on 9 November 2025). The research question was formulated using the PICO framework: Population: Human permanent teeth (in vitro, ex vivo, or in vivo) with clear aligner attachments. Intervention: Attachment removal using any technique or tool (e.g., carbide burs, polishers, fiberglass, laser, ultrasonic, magnification aids). Comparator: Alternative removal techniques or untreated enamel. Outcomes: (1) Enamel loss (µm), (2) Residual composite (%) or area, (3) Surface roughness/gloss, (4) Removal time.

The research question was “Do removal techniques, instruments, operator experience, and magnification aids influence enamel loss and residual composite following clear aligner attachment removal?”

### 2.2. Search Strategy

A comprehensive electronic search was conducted across multiple scientific databases, including PubMed, Embase, Scopus, Web of Science, and Scielo, to identify studies evaluating clear aligner attachment removal and its effects on enamel integrity. The search encompassed publications up to 25 October 2025. Additional searches were performed in Google Scholar and the reference lists of relevant articles to ensure exhaustive identification of potentially eligible studies. No language restrictions or filters (such as date, study design, or human teeth only) were applied. The search strategy aimed to retrieve studies assessing enamel loss, residual composite, surface roughness, and procedural outcomes associated with different attachment removal techniques. The following search string was applied in PubMed and adapted to other databases (Table 1).

In addition to electronic searches, manual screening of reference lists from included studies and relevant reviews was performed to maximize retrieval of eligible studies.

### 2.3. Study Selection

Two independent reviewers (R.B., A.H.) screened all retrieved titles, abstracts, and full texts against predefined inclusion and exclusion criteria. Studies were considered eligible if they (1) involved human permanent teeth with clear aligner attachments in in vitro, ex vivo, or in vivo settings; (2) evaluated attachment removal using any instrument or technique compared to alternative methods or untreated enamel; and (3) reported quantitative outcomes, including enamel loss, residual composite, surface roughness, or removal time. Case reports, reviews, animal studies, thesis, and studies with insufficient or non-extractable data were excluded. Any discrepancies between reviewers were resolved through discussion or consultation with a third reviewer.

### 2.4. Data Extraction

Data were independently extracted by two reviewers (R.B., N.N.) from each included study using a standardized collection form. Extracted information encompassed study characteristics (first author, year, study design, and sample size), tooth or substrate type, composite or attachment material, removal protocols and instruments, operator experience, use of magnification aids, and reported outcomes. Key findings were summarized narratively, highlighting trends, consistencies, and variations in enamel loss, residual composite, surface roughness, and procedural efficiency across studies.

### 2.5. Quality Assessment

The risk of bias of the included in vitro studies was independently assessed by two reviewers using a modified Joanna Briggs Institute (JBI) critical appraisal checklist for laboratory studies [24]. The checklist was adapted to reflect laboratory-based study designs, particularly regarding specimen preparation standardization, operator-dependent procedures, and measurement reproducibility.

Four domains were evaluated: D1—study planning and allocation; D2—sample preparation and standardization; D3—outcome assessment; and D4—data analysis and reporting. Domain D1 included criteria such as the use of appropriate control groups, randomization of specimens, and justification of sample size. Domain D2 focused on the standardization of substrates, composite application, and instrument calibration. Domain D3 assessed reproducibility of measurements, consistency of protocols, and implementation of operator blinding. Domain D4 evaluated the appropriateness of statistical analyses and the clarity of outcome reporting.

Each criterion was rated as ‘sufficiently reported,’ ‘insufficiently reported,’ ‘not reported,’ or ‘not applicable.’ Item-level ratings were interpreted as indicators of low (sufficiently reported), unclear (insufficiently reported), or high (not reported) risk of bias. Discrepancies between reviewers were resolved through discussion or by involving a third reviewer until consensus was reached.

A study was judged as low risk of bias when most domains were sufficiently reported with no critical omissions; moderate risk of bias when one or more domains were insufficiently reported but no serious methodological flaws were identified; and high risk of bias when multiple key domains were insufficiently reported or not reported. Detailed item-level judgments are presented in Supplementary Table S2.

### 2.6. Certainty of Evidence

The certainty (confidence) in the body of evidence for each outcome was assessed using a structured qualitative framework conceptually informed by the principles of the GRADE approach, adapted for in vitro and ex vivo studies. Because GRADE is primarily designed for clinical intervention research, a modified evaluation strategy was employed.

Certainty judgments were based on five predefined domains:

(1) Risk of bias (derived from the modified JBI appraisal), (2) consistency of findings across studies, (3) directness of the evidence relative to clinical conditions, (4) precision of reported quantitative measurements (e.g., variability and completeness of enamel loss data), and (5) methodological heterogeneity among studies.

Evidence certainty was categorized as high, moderate, or low based on the cumulative assessment of these domains. Outcomes supported by multiple studies with consistent findings, low or moderate risk of bias, and clearly reported quantitative data were considered higher certainty. Conversely, outcomes derived from single studies, inconsistent findings, imprecise measurements, or methodological limitations were downgraded. Two independent reviewers (R.B., N.N.) conducted the certainty assessment. Disagreements were resolved through discussion or consultation with a third reviewer.

Given the limited number of studies (n = 3), their exclusively laboratory-based design, and methodological heterogeneity, overall certainty was generally considered low to moderate, and conclusions were interpreted as exploratory and hypothesis-generating.

## 3. Results

### 3.1. Study Selection and Flow Diagram

**Figure 1:**
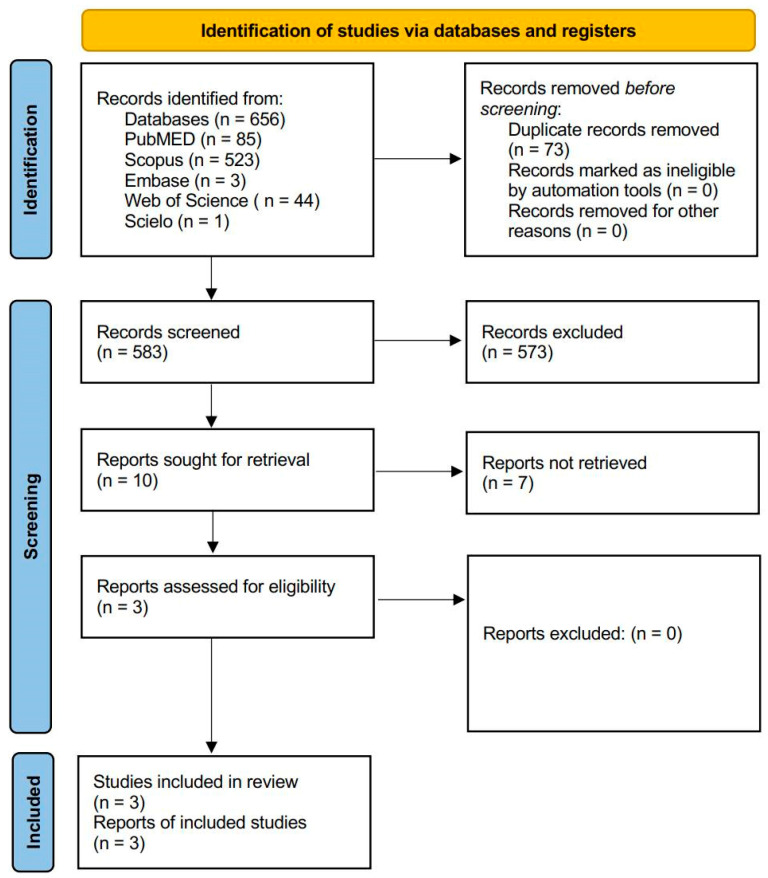
PRISMA flow diagram of study selection.

The systematic search identified a total of 656 records across the selected databases. After removal of duplicates, 583 records were screened by title and abstract. Of these, 573 records were excluded. Ten reports were sought for retrieval, of which seven could not be retrieved. The remaining three full-text articles were assessed for eligibility. All three studies met the predefined inclusion criteria and were included in the qualitative synthesis (Figure 1). All included studies were conducted in vitro or ex vivo between 2023 and 2025 and evaluated enamel loss, surface roughness, and residual composite following various clear aligner attachment removal protocols. The PRISMA 2020 checklist is presented separately in Supplementary Table S1.

### 3.2. Study Characteristics

The main characteristics of the included studies are summarized in Table 2. The studies collectively investigated different removal instruments and protocols, including traditional thermoformed trays, Computer-Aided Design and Computer-Aided Manufacturing (CAD-CAM) positioners, carbide burs, fiberglass, polishing kits, and magnification aids. Outcomes measured included enamel loss (µm), residual composite coverage (%), surface roughness, and procedural time.

### 3.3. Qualitative Analysis of Included Studies

The three in vitro studies included in this systematic review collectively investigated the efficacy, precision, and enamel preservation potential of various attachment removal techniques following clear aligner therapy. Despite methodological heterogeneity, all studies shared the overarching objective of identifying protocols that minimize enamel loss while ensuring complete removal of residual composite.

Vandeloise (2024) [25] evaluated two dedicated attachment removal systems—Smoozies (Komet, Paris, France) and Easycomp (Easycomp RA, EY-1, Eve, Keltern, Germany)—using three-dimensional profilometry and digital microscopy for quantitative surface characterization. The mean enamel loss was 22.7 ± 29.4 µm, and the average residual composite coverage was 34.4%, which was notably reduced with the adjunctive use of 2.5× magnification loupes. The use of magnification enhanced the operator’s ability to differentiate enamel from composite material, thereby improving removal precision.

Hilal Turkoglu and Atik (2025) [22] compared the enamel effects of different rotary instruments—fiberglass burs (Morelli, Brazil) and 12- and 24-blade carbide burs (Frank Dental, Germany)—applied sequentially with Renew polishing stones (Renew Finishing System; Reliance Orthodontic Products Inc., IL, USA) following attachment removal. Using three-dimensional optical profilometry, micro-computed tomography, and Geomagic software analysis, they demonstrated that fiberglass burs produced smoother enamel surfaces but were associated with greater enamel loss. In contrast, carbide burs resulted in rougher surface textures while preserving more enamel substrate. No statistically significant differences in residual composite were observed between the groups.

Nguyen and Nguyen (2025) [26] performed a comprehensive comparison of OneGloss, Enhance, SM104, Sof-Lex discs, tungsten carbide burs, zirconia burs, and white stone burs. Enamel surface topography was analyzed using surface profilometry, three-dimensional scanning, and scanning electron microscopy. White stone burs resulted in the highest enamel roughness and greatest material loss. In contrast, OneGloss, Enhance, and SM104 systems removed composite within 45–50 s and were associated with lower enamel roughness. Sof-Lex discs and zirconia burs produced relatively smooth surfaces but required longer application times.

Across the included studies, reported enamel loss ranged approximately from 15 µm to greater than 50 µm, while residual composite coverage varied between approximately 20% and 40%. Collectively, these findings reflect an increasing emphasis on minimally invasive and precision-controlled attachment removal protocols. The integration of multistep polishing systems, magnification devices, and standardized digital measurement methods was associated, within the included laboratory investigations, with improved enamel preservation and procedural reproducibility. However, interstudy variability in sample preparation, measurement calibration, outcome definitions, and operator experience precludes direct quantitative comparison or meta-analysis. Future investigations should adopt standardized profilometric parameters, calibrated enamel reference areas, and blinded outcome assessment to enhance methodological rigor and support the development of evidence-based clinical recommendations.

### 3.4. Quality Assessment and Risk of Bias

The methodological quality of the included studies was assessed using a modified Joanna Briggs Institute (JBI) critical appraisal checklist adapted for in vitro and ex vivo investigations. The checklist was selected due to its structured evaluation of methodological rigor and was adapted to reflect laboratory-based study designs, incorporating domains related to specimen preparation standardization, operator-dependent procedures, and measurement reproducibility.

Each study was evaluated across four domains: planning and allocation (D1), sample/specimen preparation (D2), outcome assessment (D3), and data treatment and reporting (D4). Individual items were rated as low risk, high risk, or unclear risk of bias based on predefined operational criteria. Overall study quality was determined according to the distribution of item-level judgments across domains.

The risk-of-bias evaluation for the included studies is summarized in Table 3. In Domain D1 (planning and allocation), all studies clearly described their removal protocols and included appropriate control comparisons (Item 1.1). However, sample randomization (Item 1.2) was inconsistently reported, and sample size justification (Item 1.3) was insufficient in most cases, indicating potential selection bias. In Domain D2 (sample/specimen preparation), all studies demonstrated adequate standardization of tooth selection, embedding, and procedural parameters (Item 2.1), while maintaining uniform experimental conditions (Item 2.2), reflecting strong methodological control. In Domain D3 (outcome assessment), measurement methodologies such as 3D profilometry, scanning electron microscopy, and micro-computed tomography (Item 3.1) were thoroughly described. However, operator blinding (Item 3.2) was not implemented in any study, introducing potential detection bias. In Domain D4 (data treatment and outcome reporting), all studies applied appropriate statistical analyses (Item 4.1) and reported data transparently (Item 4.2), supporting analytical reliability.

Overall, Nguyen and Nguyen (2025) [26] fulfilled all criteria and was rated as low risk of bias across domains. Vandeloise (2024) [25] and Hilal Turkoglu and Atik (2025) [22] were categorized as moderate risk of bias due to insufficient reporting of randomization, operator blinding, and sample size justification. Despite these limitations, methodological quality was considered acceptable for qualitative synthesis. The adapted checklist and detailed item-level judgments are provided in Supplementary Table S2.

### 3.5. Outcomes and Narrative Certainty

The included studies reported that instrument type and magnification aids were associated with differences in enamel loss, surface roughness, residual composite, and removal time following clear aligner attachment removal. Aluminum oxide-based one-step polishing systems were associated with minimal enamel loss and shorter removal times under laboratory conditions, whereas fiberglass and white stone burs were associated with increased enamel loss or surface roughness.

Certainty of evidence ranged from low to moderate across outcomes. This grading reflects the small number of available studies (n = 3), their exclusively laboratory-based design, methodological heterogeneity, and moderate risk of bias identified in two of the included studies (Table 4).

## 4. Discussion

This systematic review sought to assess how the removal of clear aligner attachments impacts enamel surfaces and residual composite, focusing on the influence of removal techniques, types of instruments used, operator experience, and the use of magnification aids on both enamel loss and remaining composite. The three in vitro studies included in this systematic review collectively examined the efficacy, precision, and enamel-preserving potential of various attachment removal techniques following clear aligner therapy [22,25,26]. Despite methodological differences, all studies shared the overarching goal of identifying protocols that minimize enamel loss while ensuring complete removal of residual composite. Given that all included studies were in vitro/ex vivo investigations, the findings should be interpreted as exploratory and hypothesis-generating rather than directly translatable to clinical practice.

Vandeloise (2024) [25] suggested that magnification improved visual discrimination between enamel and composite, thereby enhancing procedural precision and minimizing iatrogenic enamel damage. These results underscore the importance of operator visibility in achieving high-quality outcomes and highlight the potential of magnification-assisted techniques to optimize both safety and efficiency during attachment removal [25]. The findings indicate that the removal of clear aligner attachments can lead to measurable enamel loss and residual composite, consistent with prior evidence from orthodontic debonding studies reporting similar iatrogenic effects on enamel surfaces. The extent of enamel damage appears to depend on the removal protocol employed within laboratory conditions, and any extrapolation to clinical practice should be considered hypothesis-generating. Current recommendations suggest using a tungsten carbide bur for bulk composite removal, followed by silicone polishers to eliminate the final layer of resin cement, thereby minimizing enamel alteration [11,13,15,16,27,28,29,30,31].

The enamel loss observed across included studies ranged from approximately 15 µm to over 50 µm, a magnitude that, while clinically moderate, is relevant given that enamel thickness is non-regenerative [22,25,26]. While this provides an empirical estimate under laboratory conditions, the clinical significance remains uncertain. These results corroborate those reported by Vandeloise (2024) [25], who observed mean enamel loss values of 22.7 ± 29.4 µm after clear aligner removal, confirming that even conservative protocols can induce measurable surface alteration [25].

The review highlights that the type of removal instrument and visualization method are decisive factors influencing enamel preservation and surface quality. Tungsten carbide burs, followed by silicone polishers, provided the best balance between removal efficiency and enamel integrity, in agreement with multiple studies on bracket debonding where carbide burs were reported to be less aggressive compared to diamond burs or airborne abrasion techniques [28,29,30,31,32,33]. However, protocols relying solely on silicone polishers, while yielding smoother enamel surfaces, tended to leave greater amounts of residual composite, consistent with Vandeloise’s findings showing a 34.4% composite coverage, more frequent with silicone polishers than with carbide burs [25]. Residual composite resin cement on the tooth surface after attachment removal also remains a concern, as these remnants can undergo aging and shade alteration over time, potentially affecting aesthetics [34]. These are plausible considerations in vitro, but the included studies did not assess long-term clinical outcomes.

A major determinant of outcome quality identified by Vandeloise (2024) [25] was operator visibility, particularly using magnification loupes (≥2.5×). The adjunctive use of magnification significantly reduced residual composite and improved procedural precision [25], a finding supported by Baumann et al. [15], who demonstrated that magnification enhances discrimination between enamel and composite during orthodontic bracket removal. Moreover, Bernard established that the human eye can only distinguish features larger than approximately 50 µm, reinforcing the necessity of optical enhancement for complete composite removal [35]. Modern magnification systems with adequate optical clarity and ergonomic design may improve visualization and operator posture, potentially facilitating more precise differentiation between enamel and residual composite during intricate procedures. Nevertheless, controlled clinical studies are required to determine the extent to which magnification influences enamel preservation and procedural efficiency under real clinical conditions.

Another point of interest is the inverse relationship between enamel loss and residual composite. Surfaces with remaining composite often exhibited reduced measured enamel loss, as residual resin masked part of the enamel in profilometric analyses. Thus, while less enamel loss may appear favorable, it can indicate insufficient removal of resin, which may have long-term aesthetic and biological implications. Residual composite can undergo discoloration and surface degradation over time [34,36], impairing aesthetics and promoting biofilm accumulation and caries formation [34,37,38]. These outcomes remain speculative within the context of in vitro studies.

Regarding procedure duration, Vandeloise (2024) reported that magnification also tended to reduce working time [25], contrary to the findings of Mohebi et al. [21], though the difference was not statistically significant. Similarly, Soares Tenório et al. [19], observed shorter working times when using tungsten carbide burs compared to silicone polishers. However, as tungsten carbide burs can be more aggressive toward enamel, their use should be limited to bulk composite removal, followed by polishing with silicone instruments under magnification, a strategy that this review suggests could represent a balanced and potentially safer approach under laboratory conditions. A limitation noted in Vandeloise’s study [25] is the potential operator-related bias. The involvement of multiple operators with differing experience levels and habits regarding magnification use may have introduced variability in outcomes. Operators accustomed to magnification may find it difficult to perform adequately without it, and vice versa. These limitations further highlight that observed effects are exploratory and laboratory specific. Despite this, the data consistently indicate that magnification-assisted protocols provide superior control, precision, and safety compared to unaided vision. Overall, the collective evidence from this review and Vandeloise’s experimental findings [25] suggests that sequential carbide–silicone polishing under magnification may represent a promising approach under laboratory conditions.

Hilal Turkoglu and Atik (2025) [22] evaluated how different rotary instruments influence enamel integrity following attachment removal. Their findings contribute to the broader understanding that instrument selection directly affects the balance between surface smoothness and enamel preservation within the in vitro context [22]. Under laboratory conditions, fiberglass burs produced smoother enamel surfaces but were associated with greater enamel loss, whereas carbide burs preserved more enamel at the expense of increased surface roughness. Importantly, no significant differences were detected in residual composite among instruments, suggesting comparable cleaning efficacy in vitro. Moreover, these findings emphasize that operator-related factors, including applied pressure, angulation, hand stability, experience, and familiarity with magnification, can substantially influence enamel outcomes. Variability in these parameters may lead to differing degrees of enamel loss or residual composite even when standardized protocols are applied, highlighting the need for operator calibration and training. However, given the in vitro design, these observations should be interpreted cautiously and warrant clinical validation.

The observation that over 50% of the total attachment volume was removed by the plier, regardless of composite viscosity, underscores the practicality of using pliers as an initial step. This mechanical removal reduces the need for extended rotary instrumentation, potentially lowering aerosol production and minimizing operator exposure, as previously emphasized by Eliades et al. [39]. Furthermore, the independence of composite type (flowable vs. packable) suggests that clinicians can select materials based on handling preference without significantly influencing removal efficiency [22]. In contrast, packable composites exhibited higher post-attachment removal roughness, particularly after the tungsten carbide bur protocol, due to their higher filler content and viscosity. These materials demand more contact pressure during removal, increasing the likelihood of surface abrasion. This agrees with Cesur et al. [40] and Paolone et al. [41], who both demonstrated that composites with higher filler loading are more resistant to removal and can lead to greater enamel alterations when inappropriate bur types or excessive force are applied.

When comparing fiberglass burs with tungsten carbide burs followed by Renew polishing, this study found that fiberglass-based finishing produced smoother enamel but also a greater volume and area of demineralization on micro-CT evaluation. This could be explained by the fact that fiberglass burs, while yielding better surface topography, may induce deeper structural enamel loss. The explanation may lie in the bur composition: the flexible glass fibers generate micro-cutting and polishing action that can remove subsurface enamel layers not readily detected in two-dimensional (2D) profilometric evaluation but visible through 3D volumetric analysis [22]. This phenomenon is consistent with the systematic review of Paolone et al. [41], where enamel volumetric loss after adhesive removal ranged between 0.02 ± 0.01 mm^3^ and 0.61 ± 0.51 mm^3^ per tooth. The micro-CT results of the present study showed enamel loss values within this range, confirming the clinical acceptability of both tested methods. Importantly, no significant differences in demineralization depth were observed between the packable composite groups, suggesting that the wear induced by both protocols remained within a physiologically tolerable threshold.

Interestingly, although fiberglass burs generated a smoother enamel, micro-CT revealed higher volumetric enamel loss compared to carbide burs. This apparent contradiction highlights the limitation of relying solely on 2D surface parameters and underlines the importance of 3D imaging modalities such as micro-CT for a complete assessment of enamel integrity. These results also corroborate Thys et al. [42], who demonstrated that multi-step polishing systems could induce additional enamel abrasion despite improved smoothness values. This sequence effectively minimizes enamel loss while optimizing surface finish. Nevertheless, given the in vitro design of this study, intraoral factors such as salivary flow, pH fluctuations, and thermal changes were not simulated, and these may influence both adhesion and enamel response during attachment removal [43]. Therefore, in vivo studies are needed to validate these laboratory-based results.

Nguyen and Nguyen (2025) [26] evaluated seven one-step composite attachment removal systems to determine their impact on enamel surface integrity following clear aligner therapy. To our knowledge, it is the first study to specifically assess these tools for attachment removal and to propose a risk–benefit model that considers both procedural efficiency and enamel preservation [26]. These results demonstrated that aluminum oxide-based one-step systems (One-Gloss, Enhance, and SM 104) provided a suitable balance between enamel preservation and clinical efficiency. These systems achieved composite removal within 60 s while maintaining surface roughness and enamel loss at minimal levels, aligning with previous reports highlighting the enamel-friendly performance of OneGloss and Enhance polishers [44,45]. Despite no statistically significant differences among these systems in profilometric parameters, SEM analysis revealed that OneGloss and SM 104 achieved the smoothest surfaces, characterized by shallow, parallel grooves without crater formation. This corroborates prior findings emphasizing the superior finishing ability of single-step aluminum oxide-based systems compared to tungsten carbide burs [46,47]. The white stone bur, although the fastest tool tested, induced the highest surface roughness and enamel loss. These findings align with the aggressive abrasive nature reported previously [48]. SEM images revealed extensive crater formation and deep scratches, reflecting irreversible enamel damage. Such alterations are clinically relevant since the outer enamel layer is highly mineralized and provides the primary defense against acid attack and dentin hypersensitivity [49]. Furthermore, roughened enamel surfaces are known to promote bacterial adhesion, particularly when surface roughness exceeds 0.2 μm [50,51], increasing the risk of plaque accumulation and secondary caries. Consequently, despite its procedural speed, the white stone bur was associated with greater enamel damage and therefore may not be preferable when enamel preservation is prioritized.

Contrary to the findings of Shah et al. [47] and Almudhi et al. [52], where Sof-Lex discs exhibited superior smoothness when applied in multi-step protocols, our single-step Sof-Lex application did not significantly differ from the OneGloss or Enhance systems in surface roughness outcomes. These discrepancies highlight the strong operator dependency of enamel polishing outcomes. Variations in applied pressure, angulation, and duration can substantially influence results, even with standardized protocols. To minimize such variability, all procedures in this study were conducted by a single calibrated operator under controlled parameters. Nonetheless, operator technique remains a crucial variable, and future studies should further evaluate inter-operator variability in enamel surface outcomes.

The risk–benefit matrix may serve as a conceptual framework for comparing procedural trade-offs under laboratory conditions. The matrix revealed that OneGloss and SM 104 provided the most favorable outcomes, combining short removal times with minimal enamel damage, whereas the white stone bur, although efficient, represented a high-risk tool due to extensive surface abrasion. Furthermore, the previous literature highlights that pre-existing enamel alterations, such as bleaching, can affect bonding strength and susceptibility to damage during debonding [53], supporting the relevance of enamel condition in evaluating removal techniques. Clinically, this matrix supports evidence-based decision-making by enabling individualized tool selection. For patients with thin enamel or high aesthetic demands, tools located in the low-risk quadrant (e.g., OneGloss, SM 104) should be prioritized. Conversely, in time-sensitive clinical scenarios, such as pediatric treatments, tools offering faster removal with moderate enamel impact may be considered [26].

This study’s strengths include the standardization of attachment size and material, ensuring reproducibility, and the use of high-resolution 3D scanning and profilometry, which provide quantitative precision beyond traditional ARI-based assessments [5,6]. Nevertheless, certain limitations should be noted. The study did not account for the effects of different composite viscosities, which may influence removal difficulty and enamel damage. Packable composites, with their higher filler content and viscosity, may require increased mechanical effort during removal, potentially increasing roughness and loss [54]. Furthermore, aging effects of intraoral conditions, such as temperature variation, salivary pH, and microbial activity, were not simulated, potentially underestimating enamel loss compared to clinical reality [55,56]. Additionally, the study relied on SEM for surface evaluation, a two-dimensional method that cannot fully characterize topographical depth variations. Future studies should incorporate 3D imaging modalities, such as atomic force microscopy (AFM), confocal laser microscopy, or optical profilometry, to enhance surface characterization accuracy [57]. Finally, clinicians should also consider aerosol safety during composite removal. Eliades and Koletsi [39] emphasized the risk of airborne nanoparticle release during grinding. Since clear aligner attachments typically have larger surface areas than bracket adhesives, the risk of aerosol generation is higher, underscoring the importance of using high-volume suction and appropriate PPE during debonding. Among the seven evaluated one-step removal systems, OneGloss and SM 104 provided the most favorable combination of time efficiency, surface smoothness, and enamel preservation, restoring the enamel to near pre-treatment conditions. The risk–benefit matrix proposed in this study offers a practical decision-making tool for clinicians to balance procedural efficiency with enamel safety. Further research integrating different composite types, aging conditions, and advanced 3D surface analysis is recommended to expand these findings and optimize clinical protocols for clear aligner attachment removal [26].

The present systematic review included only three in vitro/ex vivo studies, which inherently limits the generalizability of the findings to real clinical settings. The small number of available studies reflects the novelty of this research topic and underscores the current lack of high-quality evidence concerning enamel alterations and residual composite following the removal of clear aligner attachments. Accordingly, the conclusions of this review should be interpreted as exploratory rather than confirmatory. Furthermore, methodological heterogeneity among studies, including differences in measurement techniques (SEM, profilometry, micro-CT), substrate preparation (intact vs. previously restored enamel, etching protocols), composite types (flowable vs. packable, filler content, viscosity), removal protocols (bur type, sequence, applied pressure, duration), and operator-related factors (experience, magnification use, hand stability), precluded quantitative synthesis through meta-analysis. The absence of standardized protocols for measuring enamel loss and residual composite coverage also introduces potential bias, complicating interstudy comparisons. Moreover, none of the included studies assessed long-term outcomes, such as susceptibility to plaque accumulation, discoloration, or enamel remineralization potential following attachment removal. The limited data on operator magnification use and ergonomic factors further restricts insight into their clinical impact. Adding, an important methodological limitation across all included studies was the absence of operator blinding and the lack of standardization regarding operator experience and familiarity with magnification devices. Given that composite removal and enamel polishing are technique-sensitive procedures, operator skill and visual training may substantially influence outcomes. The absence of calibration or crossover designs introduces potential performance bias, particularly in studies comparing magnification-assisted versus unaided protocols. Consequently, the reported advantages of magnification must be interpreted with caution, as they may partially reflect operator-related factors rather than the optical aid itself. In addition to the small number of included studies and their exclusively laboratory-based design, all investigations lacked operator blinding and did not control for operator experience or familiarity with magnification systems. These factors represent potential sources of systematic error and may have influenced enamel loss measurements, residual composite detection, and procedure duration. Particularly for magnification-assisted protocols, the absence of standardized operator calibration limits the ability to attribute observed differences solely to the intervention rather than to performance bias.

It is essential to distinguish between surface roughness and volumetric enamel loss, as these parameters describe fundamentally different aspects of enamel alteration. Surface roughness (e.g., Ra, Rz, Sa) reflects micro topographical irregularities of the enamel surface and is primarily associated with aesthetic appearance and potential plaque retention [58]. In contrast, volumetric enamel loss quantifies the actual reduction in enamel thickness or volume and represents irreversible structural tissue removal. A surface may exhibit low roughness yet still demonstrate significant volumetric enamel loss, and conversely, increased roughness does not necessarily imply substantial structural loss. Therefore, these outcomes should not be interpreted as interchangeable measures of enamel damage. Future studies should avoid conflating surface roughness with enamel loss and should report both parameters independently to allow biologically meaningful interpretation including meta-analysis.

Despite the limited number of studies, the collective evidence suggests that clear aligner attachment removal can cause measurable enamel loss (≈15–50 µm) and residual composite (≈20–40% of surface area), both of which depend on the removal technique and visualization method used. Sequential carbide–silicone polishing systems, especially when used under ≥2.5× magnification, demonstrated the most favorable outcomes, achieving effective composite removal while minimizing enamel damage. In contrast, fiberglass and white stone burs, though efficient in removal speed, were associated with increased enamel roughness and surface irregularities. Alternative methods were explored such as laser-assisted removal, but their efficacy and safety require further investigation. These findings highlight the importance of balancing removal efficiency with enamel preservation, emphasizing that controlled, stepwise protocols using minimally abrasive systems under magnification offer the best clinical compromise.

Future research should prioritize standardized, clinically oriented protocols that simulate intraoral conditions, including variables such as salivary contamination, thermal cycling, and enamel curvature. Incorporating in vivo or ex vivo designs with larger sample sizes, diverse composite formulations, and different magnification aids would enhance external validity. Advanced imaging modalities such as 3D optical profilometry, atomic force microscopy (AFM), or confocal laser scanning microscopy (CLSM) should be integrated to provide more precise quantification of surface topography and volumetric enamel loss. Furthermore, clinical trials comparing operator experience levels and tool ergonomics would help translate laboratory findings into practical recommendations.

## 5. Conclusions

Within the limitations of the available evidence, removal of clear aligner attachments was associated with measurable enamel loss (approximately 15–50 µm) and residual composite coverage under laboratory conditions, with outcomes influenced by removal technique, instrument selection, and operator visibility. Sequential carbide–silicone polishing protocols performed under magnification were reported to be associated with reduced residual composite and controlled enamel alteration in the included in vitro/ex vivo studies. However, given the small number of studies, their laboratory-based design, and methodological heterogeneity, these findings should be interpreted as exploratory and hypothesis-generating rather than indicative of clinical superiority or preference. The absence of clinical trials, standardized procedural parameters, and long-term biological outcome data limits generalizability. Further well-designed experimental and clinical investigations are required to clarify these preliminary observations and to inform future protocol development.

## Figures and Tables

**Table 1:** Search strategy used in all databases screened.

Database	Search Strategy
PubMed	(“Clear Aligners”[tiab] OR “Orthodontic Appliances”[MeSH] OR “clear aligner”[tiab] OR “aligner attachment”[tiab] OR “orthodontic attachment”[tiab]) AND (“Enamel”[ tiab] OR “Dental Enamel”[MeSH] OR “enamel loss”[tiab] OR “residual composite”[tiab] OR “composite remnants”[tiab] OR “surface roughness”[tiab] OR “profilometry”[tiab] OR “SEM”[tiab]) AND (“Dental Debonding”[MeSH] OR “removal”[tiab] OR “detachment”[tiab])
Scopus	ALL (“clear aligner” OR “aligner attachment” OR “orthodontic attachment”) AND ALL (“enamel loss” OR “enamel damage” OR “residual composite” OR “composite remnants” OR “surface roughness” OR “profilometry” OR “SEM”) AND ALL (“debonding” OR “removal” OR “detachment”)
Embase	(‘clear aligner’ OR ‘aligner attachment’ OR ‘orthodontic attachment’) AND (‘enamel loss’ OR ‘enamel damage’ OR ‘residual composite’ OR ‘composite remnants’ OR ‘surface roughness’ OR ‘profilometry’ OR ‘SEM’) AND (‘debonding’ OR ‘removal’ OR ‘detachment’)
Web of Science	TS = ((“clear aligner” OR “aligner attachment” OR “orthodontic attachment”) AND (“enamel loss” OR “enamel damage” OR “residual composite” OR “composite remnants” OR “surface roughness” OR “profilometry” OR “SEM”) AND (“debonding” OR “removal” OR “detachment”))
Scielo	TS = ((“clear aligner” OR “aligner attachment” OR “orthodontic attachment”) AND (“enamel loss” OR “enamel damage” OR “residual composite” OR “composite remnants” OR “surface roughness” OR “profilometry” OR “SEM”) AND (“debonding” OR “removal” OR “detachment”))

**Table 2:** Characteristics of studies included in the systematic review.

Study & Year	Substrate	Composite/Attachment	Removal Procedures/Instruments	Methodologies	Main Outcomes	Main Results
Vandeloise, 2024 [25]	Extracted premolars/molars	Scotchbond + Filtek Supreme Ultra flowable (3M Oral Care, St. Paul, MN, USA)	Smoozies kit (Komet, Paris, France), Easycomp kit (Easycomp RA, EY-1, Eve, Keltern, Germany), 2.5× magnification loupes	3D profilometry, digital microscopy	Enamel loss (µm), Residual composite (%), Procedure time (s)	Mean enamel loss: 22.7 ± 29.4 µm; Residual composite: 34.4%; Reduced residual composite with loupes
Hilal Turkoglu & Ezgi Atik, 2025 [22]	Extracted premolars	Flowable GC Universal Injectable (GC Corporation, Tokyo, Japan) and Packable GC Genial Restorative (GC Corporation, Tokyo, Japan)	Debonding pliers; Fiberglass burs (Morelli, Sorocaba, SP, Brazil); Tungsten carbide burs (12- and 24-blade; Frank Dental, Gmund am Tegernsee, Germany); Renew polishing stone (Reliance Orthodontic Products, Itasca, IL, USA)	3D optical profilometry, micro-CT, Geomagic software	Enamel loss (µm), Surface roughness (Ra), Residual composite (%)	Fiberglass burs: smoother enamel, higher enamel loss; Carbide burs: rougher enamel, less enamel loss; No significant difference in residual composite
Nguyen & Nguyen, 2025 [26]	Extracted premolars	Escom 100 condensable composite (Spident Co., Ltd., Incheon, Republic of Korea)	OneGloss^®^ (Shofu Inc., Kyoto, Japan); Enhance^®^ (Dentsply, Milford, CT, USA); SM104 polishing system; Sof-Lex™ discs (3M Oral Care, St. Paul, MN, USA); tungsten carbide burs; zirconia burs; white stone burs	Profilometer, 3D scanning, SEM	Enamel roughness/loss, removal time, risk–benefit	White stone burs: highest roughness and enamel loss; OneGloss (Shofu Inc., Kyoto, Japan), Enhance^®^ (Dentsply, Milford, CT, USA), SM104: minimal enamel loss, low roughness, fastest removal (~45–50 s); Sof-Lex discs (3M Oral Care, St. Paul, MN, USA) and zirconia burs: smoother enamel but slower removal

**Table 3:** Quality assessment of studies included in the systematic review, according to key risk of bias domains. 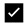
—sufficiently reported (R); 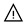
—insufficiently reported (IR); 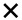
—not reported (NR).

Study	D1: Planning & Allocation			D2: Sample Preparation and Standardization		D3: Outcome Assessment		D4: Data & Reporting		Overall Risk
	1.1	1.2	1.3	2.1	2.2	3.1	3.2	4.1	4.2	
Vandeloise, 2024 [25]	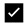	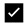	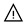	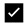	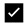	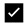	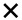	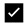	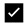	Moderate
Hilal T & Ezgi A, 2025 [22]	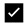	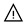	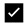	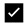	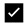	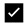	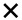	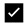	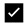	Moderate
Nguyen & Nguyen, 2025 [26]	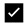	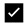	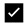	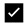	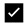	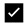	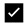	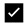	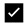	Low

Bias sources within each domain: 1.1—use of control group; 1.2—sample randomization; 1.3—justification of sample size; 2.1—standardization of materials/samples; 2.2—uniformity of experimental conditions; 3.1—consistency in testing procedures/outcomes; 3.2—blinding of the operator; 4.1—statistical evaluation; 4.2—reporting of results.

**Table 4:** Summary of outcomes and narrative certainty of evidence.

Outcome	Vandeloise, 2024 [25]	Hilal & Atik, 2025 [22]	Nguyen & Nguyen, 2025 [26]	Narrative Confidence
Enamel loss (µm)	22.7 ± 29.4	Higher with fiberglass burs, lower with carbide burs	Minimal with OneGloss^®^ (Shofu Inc., Kyoto, Japan), Enhance^®^ (Dentsply, Milford, CT, USA), SM104; higher with white stone burs	Moderate
Residual composite (%)	34.4%; reduced with loupes	No significant difference between burs	Minimal residual composite with most polishing systems	Moderate
Surface roughness/gloss (Ra)	Not reported	Smoother enamel with fiberglass burs; rougher with carbide burs	Minimal roughness with OneGloss^®^ (Shofu Inc., Kyoto, Japan)/Enhance^®^ (Dentsply, Milford, CT, USA), higher with white stone burs	Moderate
Removal time (s)	Not reported	Not reported	Fastest with OneGloss^®^ (Shofu Inc., Kyoto, Japan)/Enhance^®^ (Dentsply, Milford, CT, USA)/SM104 (~45–50 s); Sof-Lex discs (3M Oral Care, St. Paul, MN, USA) and zirconia burs slower	Low-Moderate

## Data Availability

Data sharing is not applicable. No new data were created or analyzed in this study.

## Supplementary Materials

The following supporting information can be downloaded at: https://www.mdpi.com/article/10.3390/clinpract16030051/s1, Table S1: PRISMA 2020 Checklist; Table S2: Modified JBI critical appraisal checklist for laboratory studies: Assessment of risk of bias.
