# Supplementary material for: Enamel Integrity and Residual Composite Following Clear Aligner Attachment Removal: A Systematic Review
Source: Clin Pract. 2026 Feb 27;16(3):51. doi: 10.3390/clinpract16030051 (PMC13025379; doi:10.3390/clinpract16030051)
Supplement: Supplementary file 1 [file clinpract-16-00051-s001.zip › Supplementary Table S2.pdf]

Supplementary Table S2. Modified JBI Critical Appraisal Checklist for Laboratory Studies: Assessment of Risk of Bias

Domain	Item	Description	Rating Options	Notes / Modifications for In Vitro/Ex Vivo Studies
**D1 – Study Planning and Allocation**	1.1	Was an appropriate control group used?	Sufficiently reported (R) / Insufficiently reported (IR) / Not reported (NR) / Not applicable (NA)	Maintained original JBI item; assessed presence of untreated enamel or alternative technique as control.
	1.2	Were specimens randomly allocated to groups?	R / IR / NR / NA	Adapted for lab studies: randomization of extracted teeth or standardized allocation to removal techniques.
	1.3	Was sample size justified?	R / IR / NR / NA	Modified to consider justification based on prior literature or pilot testing for in vitro/ex vivo experiments.
**D2 – Sample/Specimen Preparation and Standardization**	2.1	Were specimens prepared in a standardized manner?	R / IR / NR / NA	Included tooth type selection, embedding technique, and attachment placement protocol.
	2.2	Were experimental conditions uniform across groups?	R / IR / NR / NA	Adapted to laboratory conditions: same operator, same instruments, same magnification, and consistent environmental parameters.
**D3 – Outcome Assessment**	3.1	Were measurement methods valid and reproducible?	R / IR / NR / NA	Included SEM, 3D profilometry, micro-CT, optical scanning, or profilometry for enamel loss and residual composite.
	3.2	Was operator blinded to group allocation?	R / IR / NR / NA	Maintained original JBI item; noted that most lab studies lacked blinding but assessed attempts to minimize detection bias.
**D4 – Data Treatment and Outcome Reporting**	4.1	Were statistical analyses appropriate?	R / IR / NR / NA	Applied standard lab-based tests for enamel loss and residual composite; noted whether parametric/non-parametric tests were justified.
	4.2	Were results reported clearly and comprehensively?	R / IR / NR / NA	Adapted to include tables, figures, SEM images, or profilometry outputs; ensures transparency of enamel loss/residual composite measurements.

Overall Risk of Bias Criteria

- **Low Risk:** All or most domains sufficiently reported; no critical items missing.
- **Moderate Risk:** One or more domains insufficiently reported, but no multiple critical domains missing.
- **High Risk:** Multiple critical domains insufficiently reported or not reported.

Notes:

1. Items modified specifically for laboratory studies are italicized in the “Notes / Modifications” column.
2. This checklist guided the risk-of-bias assessment for all included in vitro/ex vivo studies in the manuscript.
3. Each study was scored per item (R / IR / NR / NA), and the overall RoB was determined according to the criteria above.
